# Vascular Heparan Sulfate and Amyloid-β in Alzheimer’s Disease Patients

**DOI:** 10.3390/ijms25073964

**Published:** 2024-04-02

**Authors:** Ilayda Ozsan McMillan, Marla Gearing, Lianchun Wang

**Affiliations:** 1 Department of Molecular Pharmacology & Physiology, Morsani College of Medicine, University of South Florida, Tampa, FL 33613, USA; iozsan@usf.edu; 2 Department of Pathology, Center for Neurodegenerative Disease, Emory University School of Medicine, Atlanta, GA 30307, USA; mgearin@emory.edu

**Keywords:** Alzheimer’s disease, heparan sulfate, amyloid-β, cerebral amyloid angiopathy, cerebrovasculature, gender, ApoE, endothelial cell, smooth muscle cell

## Abstract

Alzheimer’s disease (AD) is a debilitating neurodegenerative disease characterized by the accumulation of extracellular amyloid-β peptides (Aβ) within the cerebral parenchyma and vasculature, which is known as cerebral amyloid angiopathy (CAA). This study utilized confocal imaging to investigate heparan sulfate (HS) expression within the cerebrovasculature and its associations with Aβ, gender, and ApoE4 genotype in AD. Our investigation revealed elevated levels of HS in the cerebrovasculature of AD patients with severe CAA. Additionally, these patients exhibited higher HS colocalization with Aβ in the cerebrovasculature, including both endothelial and vascular smooth muscle cell compartments. Intriguingly, a reversal in the polarized expression of HS within the cerebrovasculature was detected in AD patients with severe CAA. Furthermore, male patients exhibited lower levels of both parenchymal and cerebrovascular HS. Additionally, ApoE4 carriers displayed heightened cerebrovascular Aβ expression and a tendency of elevated cerebrovascular HS levels in AD patients with severe CAA. Overall, these findings reveal potential intricate interplay between HS, Aβ, ApoE, and vascular pathology in AD, thereby underscoring the potential roles of cerebrovascular HS in CAA development and AD pathology. Further study of the underlying mechanisms may present novel therapeutic avenues for AD treatment.

## 1. Introduction

Alzheimer’s disease (AD) is a debilitating neurodegenerative disease that is characterized by the accumulation of extracellular amyloid-β peptides (Aβ) within the cerebral parenchyma and vasculature, which is known as cerebral amyloid angiopathy (CAA), and intracellular neurofibrillary tangles consisting of hyperphosphorylated tau [1,2,3,4]. While tau aggregation shows a stronger correlation with cognitive decline in patients, mounting evidence suggests that Aβ accumulation, which precedes tau aggregation, may serve as the major initiating event in AD pathogenesis [1]. Aβ accumulation leads to the formation of Aβ senile and neuritic plaques, which stem from misfolded Aβ peptides aggregating over time. Accumulated Aβ peptides disrupt cellular communication, trigger inflammatory responses, and ultimately lead to neuronal cell death, contributing to cognitive decline [1,2,3,4,5]. Significant efforts have been devoted to understanding the mechanisms underlying Aβ accumulation and exploring potential treatments for AD [1,2,3,4,5]. One more recent promising therapeutic avenue is anti-Aβ antibody immunotherapy, which targets and clears Aβ plaques from the brain. Antibodies like lecanemab, donanemab, aducanumab, and gantenerumab have either received FDA approval for AD treatment or are undergoing clinical trials [4,5,6,7,8,9,10]. Evidence suggests that these therapies can slow AD progression and improve cognitive function by enhancing Aβ clearance from the brain tissue [4,6,7,8,9,10]. However, it is important to note that these treatments also carry various potential side effects, including some that may be life-threatening [11]. Among the most common side effects are cerebrovascular problems, particularly amyloid imaging abnormality-related edema and hemorrhages, which can increase the risk of strokes [12,13,14]. Interestingly, anti-amyloid treatments have been associated with exacerbating CAA in some patients, suggesting a possible link between worsened CAA and the development of cerebrovascular complications in the context of anti-Aβ therapy [5]. The precise mechanisms underlying CAA development in AD and their interaction with anti-Aβ treatments remain unclear, emphasizing the need for further research in this area.

Heparan sulfate (HS) is a linear polysaccharide with various sulfation modifications, forming covalent bonds with protein cores to generate heparan sulfate proteoglycans (HSPGs) [15,16]. These HSPGs, which are distinguished by their protein cores, are found on cell surfaces and in the extracellular matrix engaging with various protein ligands. These interactions, which are mediated mainly by their HS chains, play crucial roles in regulating numerous biological processes, including organ development, angiogenesis, tumorigenesis, leukocyte trafficking, and lipid metabolism [17,18,19,20,21,22,23].

In AD patients and mouse models, HS co-deposits with Aβ in plaques within the brain tissue and blood vessels [24,25,26,27,28,29,30,31,32,33,34]. Biochemical analyses and in vitro cell studies demonstrate that HS directly binds to Aβ and accelerates Aβ aggregation [35,36,37,38,39,40]. Furthermore, HS facilitates the internalization of Aβ into cells, leading to subsequent cytotoxic effects [41,42,43,44,45]. In AD mouse models, reducing neuronal HS expression or overexpression of heparinase (HPSE), an enzyme that breaks down HS into smaller fragments, decreases brain Aβ levels [46,47,48]. These observations highlight the functional role of HS in promoting the accumulation of Aβ in the brain [46,47,48]. Interestingly, the depletion of neuronal HS or the overexpression of HPSE paradoxically exacerbates Aβ deposition in cerebral blood vessels, leading to the development or worsening of CAA. These vascular phenotypes mirror the effects of anti-Aβ treatments, suggesting a potential involvement of vascular HS in the development of CAA in AD and during anti-Aβ therapy. 

In this study, we investigated the expression of HS within the prefrontal cortex cerebrovasculature and its correlation with CAA in AD and AD risk factors, including gender and ApoE isoform.

## 2. Results

### 2.1. Cerebrovascular Aβ Accumulation in AD Patients Increases with Severe CAA

**Figure 1:**
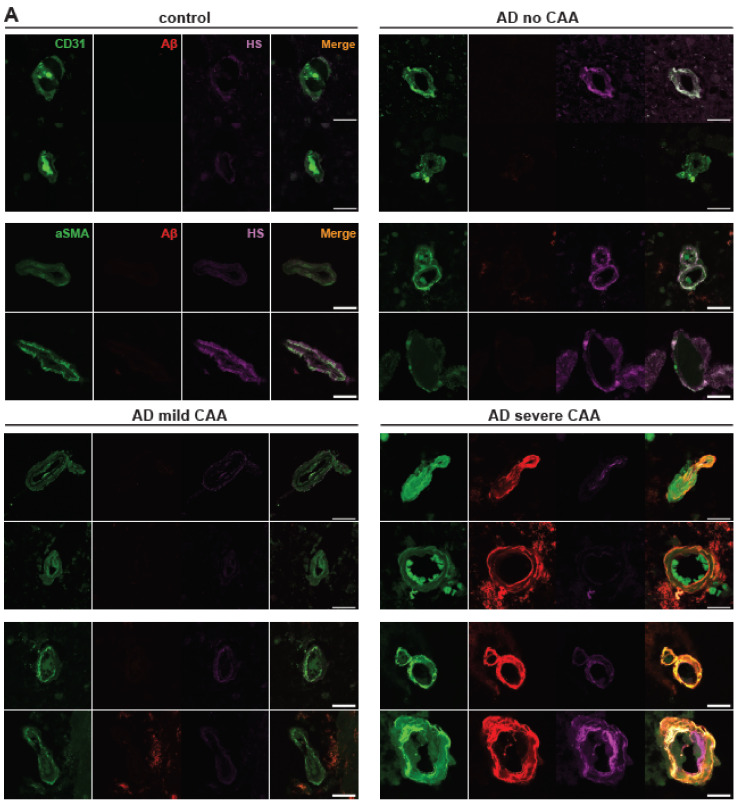

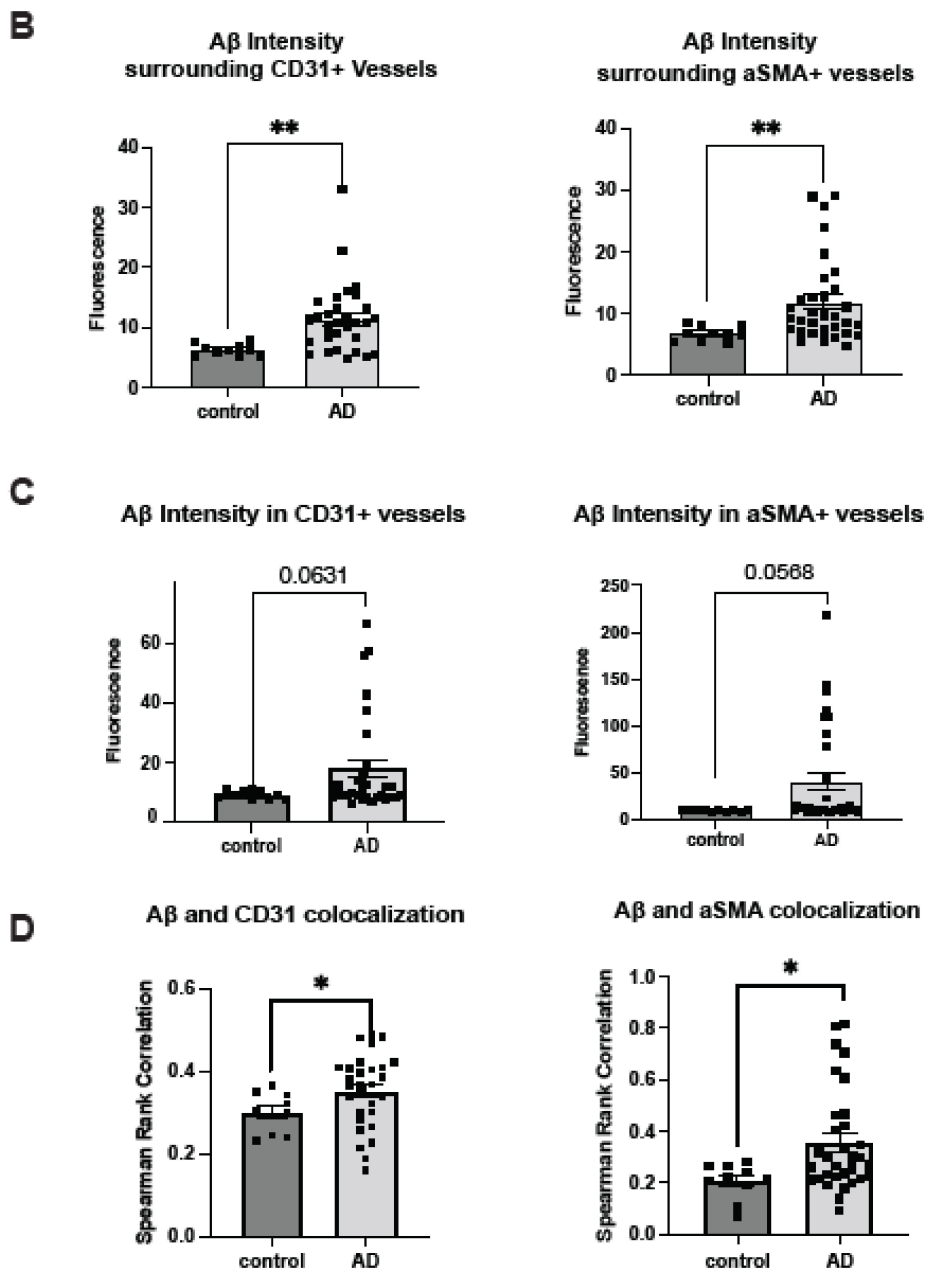

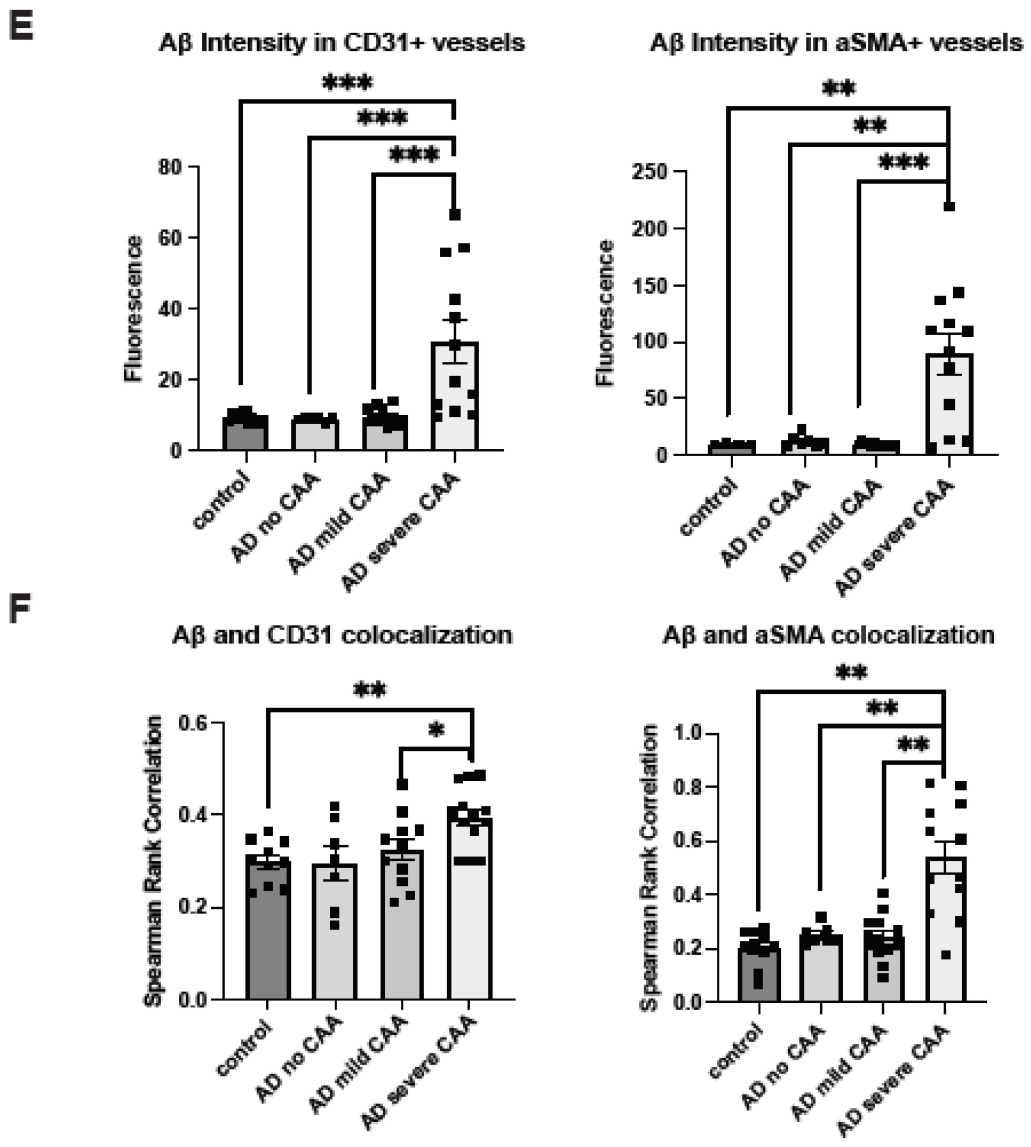
**Cerebrovascular Aβ accumulation in AD with or without CAA.** Representative immunofluorescence images staining of prefrontal cortex tissue sections of AD patients with no, mild, or severe CAA or control patients for cerebral vascular ECs (CD31+), vascular SMCs (αSMA+), total Aβ (anti-Aβ antibody D54D2), and HS (anti-HS antibody 10E4) (**A**). Aβ staining fluorescence surrounding cerebrovasculature (parenchyma) and in EC and SMC compartments were quantified. The mean levels of Aβ fluorescence are elevated in AD subjects in the prefrontal cortex parenchyma and vasculature, encompassing both ECs and SMCs ((**B**) and (**C**), respectively). The colocalization of Aβ fluorescence and vascular marker staining was assessed using Spearman’s correlation rank analysis and is notably increased in AD patients (**D**). When stratified by CAA severity in AD groups, augmented Aβ fluorescence within the vascular wall and its colocalization with CD31 or αSMA staining was not observed in patients with no or mild CAA; however, such changes are evident only in severe CAA cases, including EC and SMC compartments ((**E**) and (**F**), respectively). The data are presented as mean ± SD. The *p* values for pairwise comparisons are provided. For significance results, * = *p* ≤ 0.05, ** = *p* ≤ 0.01, *** = *p* ≤ 0.001. Scale bars = 25 µm.

Extracellular deposition of Aβ is one of AD’s most extensively studied histopathological features. In addition to its presence within the brain parenchyma, Aβ deposition also occurs within the cerebrovasculature, leading to CAA in up to 98% of AD patients [1,5]. Aβ deposition was assessed using the pan anti-Aβ antibody D54D2 in conjunction with anti-CD31 staining for ECs or anti-αSMA staining for vascular SMCs (Figure 1A). The average intensity of Aβ staining fluorescence was quantified to reflect Aβ deposition within the stained regions and their adjacent parenchyma. In the parenchyma surrounding cerebrovasculature in AD patients, the mean Aβ deposition was significantly elevated around ECs compared to controls (control mean = 6.33, AD mean = 11.46, Z = −2.98292, *p* = 0.0029), as well as in the vicinity of SMCs (control mean = 6.81, AD mean = 11.82, Z = −2.83511, *p* = 0.0046) (Figure 1B), thereby confirming Aβ accumulation in the parenchyma of AD patients. Mean vascular Aβ levels showed an upward trend in AD patients (Figure 1C), although statistical significance was not reached in CD31+ area (control mean = 9.21, AD mean = 17,82, Z = −1.85847, *p* = 0.0631) and αSMA+ area (control mean = 9.57, AD mean = 40.55, Z = −1.90476, *p* = 0.0568).

To gain a more comprehensive understanding of Aβ deposition in the cerebrovasculature, we conducted a quantification of the vascular area with Aβ deposition in AD, which was performed through Aβ and CD31+ or αSMA+ colocalization analysis. Notably, CD31 fluorescence and Aβ fluorescence colocalization significantly increased in patients with AD (control mean = 0.30, AD mean = 0.35, Z = −2.01, *p* = 0.044) in Spearman’s correlation rank analysis (Figure 1D). In Manders’ coefficients colocalization analysis, Manders’ M2 exhibited a significant increase in Aβ colocalization with CD31 in AD patients. At the same time, M1 indicated that CD31 colocalization with Aβ remained unchanged (Figure S1A). In SMCs, a heightened colocalization of αSMA and Aβ immunofluorescence was noted in AD patients (control mean = 0.21, AD mean = 0.36, Z = −2.32, *p* = 0.0204) (Figure 1D). Manders’ coefficient analysis revealed a tendency for the colocalization of Aβ and αSMA to increase in both M1 and M2 parameters (Figure S1A). These analyses revealed elevated Aβ deposition or a trend within the cerebrovasculature of AD patients preselected based on their CAA severities, which was in agreement with their clinical CAA diagnosis.

To further characterize Aβ deposition within the cerebrovasculature in AD, we extended the analysis to Aβ deposition in AD patients categorized according to CAA severity. Upon stratifying AD samples by the extent of CAA, escalated vascular Aβ deposition exclusively emerged in AD patients with severe CAA, but not those with no or mild CAA. This is evident in the mean Aβ intensity within the CD31+ area (H(3) = 19.18, *p* = 0.0003; mean control = 9.21, mean AD no CAA = 8.83, mean AD mild CAA = 9.51, mean AD severe CAA = 30.63) and αSMA+ area (H(3) = 17.92, *p* = 0.0005; mean control = 10.15, mean AD no CAA = 12.59, mean AD mild CAA = 9.74, mean AD severe CAA = 90.25) (Figure 1E). As expected, marked increases in vascular Aβ were observed in AD patients with severe CAA when compared to control patients (Z = 3.53, *p* = 0.0004) and those with no CAA (Z = 3.32, *p* = 0.0009) or mild CAA (Z = 3.32, *p* = 0.0009) in the CD31+ area. Similarly, significant increases in vascular Aβ in the αSMA+ area were seen in AD patients with severe CAA when contrasted with control patients (Z = 3.26, *p* = 0.0011) and AD patients with no CAA (Z = 2.58, *p* = 0.0099) or mild CAA (Z = 3.51, *p* = 0.0005).

In the Aβ and CD31 colocalization analyses using Spearman rank correlation coefficient, Aβ and CD31 colocalization (H(3) = 12.48, *p* = 0.0059, mean control = 0.30, mean AD no CAA = 0.30, mean AD mild CAA = 0.32, mean AD severe CAA = 0.40) was higher in patients with severe CAA compared to control patients (Z = 3.40, *p* = 0.007) and AD patients with mild CAA (Z = 2.49, *p* = 0.0127) and trending compared to AD patients with no CAA (Z = 1.83, *p* = 0.068) (Figure 1F). Aβ and αSMA also significantly colocalized more in AD patients with severe CAA (H(3) = 18.24, *p* = 0.0004, mean control = 0.21, mean AD no CAA = 0.25, mean AD mild CAA = 0.24, mean AD severe CAA = 0.54) compared to control patients (Z = 3.40. *p* = 0.0007) and AD patients with mild CAA (Z = 3.40, *p* = 0.0007) or no CAA (Z = 2.83, *p* = 0.0046) (Figure 1F). In Manders’ coefficient analysis, AD patients with severe CAA showed increased Aβ colocalization with CD31 and αSMA in M1, as well as with αSMA, but not with CD31 in M2 (Figure S1B). Our immunostaining showed that increased Aβ deposition is only seen in AD patients with severe CAA. This could be because most CAA is found in the posterior cortex, and the prefrontal cortex is less affected at the mild stage (47). 

### 2.2. Cerebrovascular HS Is Elevated in AD Patients with Severe CAA

**Figure 2:**
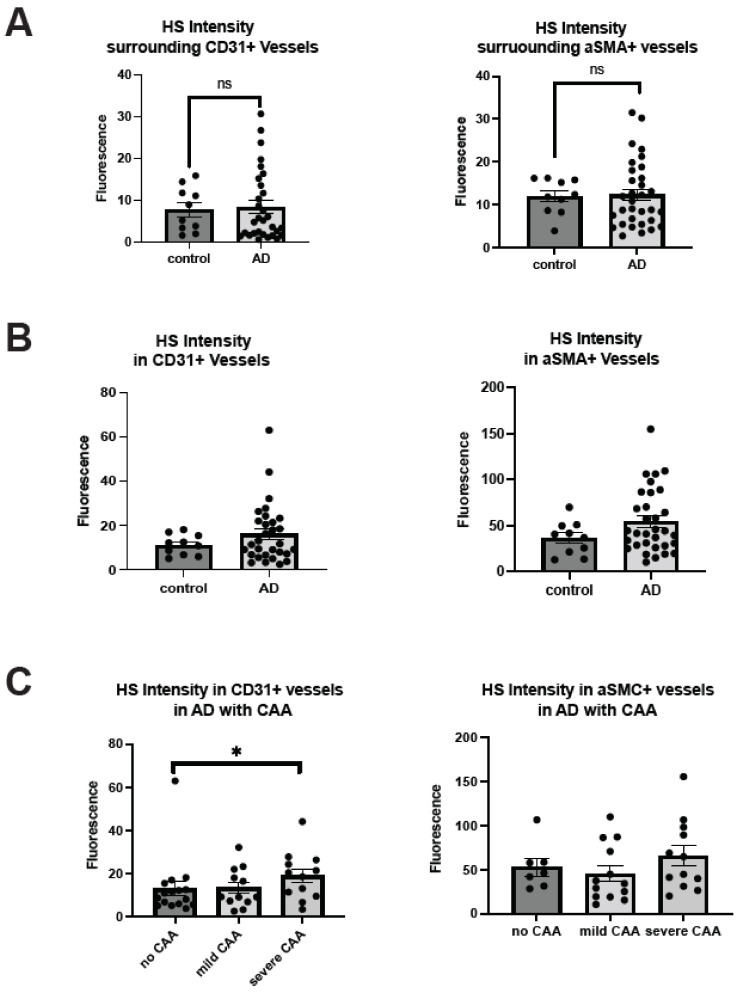

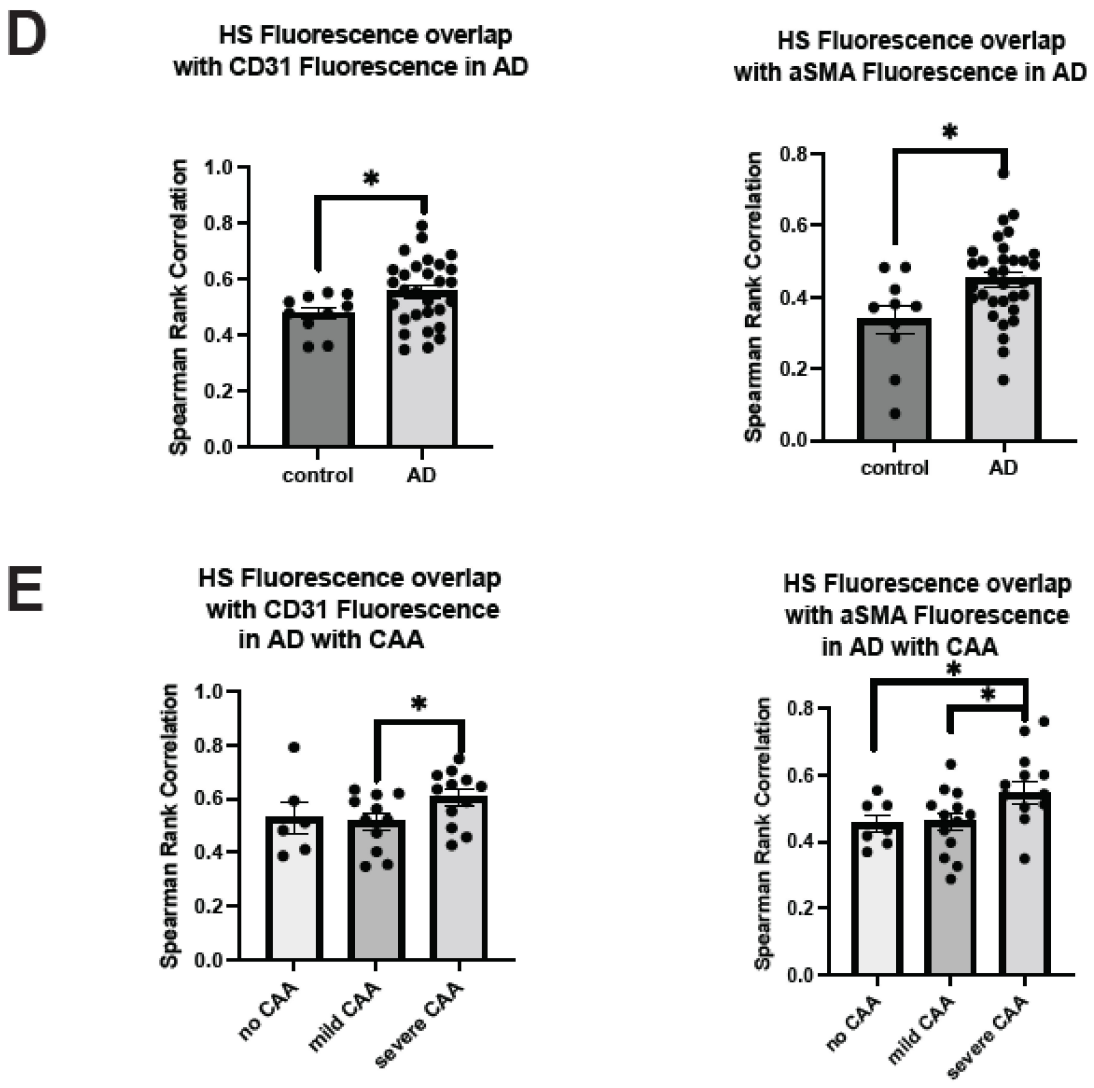
**Cerebrovascular HS in AD.** The HS densities surrounding cerebrovasculature and in the vascular EC and SMC compartments are not significantly different between AD and control patients but have a strong tendency of increased HS density in the SMC compartment ((**A**) and (**B**), respectively). In the CAA-AD subgroup analysis, vascular HS density in the EC compartment was increased in the severe CAA group, and there was a tendency for increased HS density in the SMC compartment (**C**). In co-localization analysis, HS showed significant increases in staining overlapping with CD31 and αSMA (**D**). In the CAA-AD subgroup analysis, the increased HS-CD31 and HS- αSMA staining overlap was seen only in AD patients with severe CAA (**E**). The data are presented as mean ± SD. For significance results, * = *p* ≤ 0.05; ns, not significant.

Bulk tissue analysis has determined that HS level is elevated in AD patients [24,25,49,50], but it remains unknown if HS level in cerebrovasculature is altered in the patients [33]. This was determined by the co-staining of HS and CD31 or αSMA. HS densities were not significantly changed in the parenchyma in patients with AD surrounding ECs (control mean = 7.74, AD mean = 8.30, Z = 0.48414, *p* = 0.6283) or surrounding SMCs (control mean = 6.80, AD mean = 11.82, Z = 0.32486, *p* = 0.7453) (Figure 2A). HS levels in the cerebrovasculature are also not significantly changed in AD either in EC (control mean = 11.24, AD mean = 16.07, Z = −0.67155, *p* = 0.5019) or SMC compartments (control mean = 36.49, AD mean = 54.36, Z = −1.28461, *p* = 0.1989), although there appear tendencies of increase in both compartments (Figure 2B). In the CAA-stratified subgroup analysis, the mean HS level in AD patients with severe CAA showed a significant increase in EC compartment (Z = 1.973013, *p* = 0.0485) when compared to the AD patients with no CAA (Z = 1.973013, *p* = 0.0485) (Figure 2C). Interestingly, mean HS in the SMA compartment was not significantly altered in AD patients among the CAA-stratified AD patients (H(2) = 2.6442, *p* = 0.2666), although the severe CAA group showed a tendency of increase.

In parallel, we carried out HS and vascular marker colocalization analyses. When CD31 fluorescence was examined, its overlap with HS fluorescence significantly increased in AD patients compared to control subjects (control mean = 0.4326, AD mean = 0.51, Z = −2.28, *p* = 0.001) (Figure 2D), indicating that more ECs express or are covered with above-background HS in AD. Similarly, αSMA fluorescence and HS fluorescence overlap also increased in AD patients compared to control subjects (control mean = 0.34, AD mean = 0.45, Z = −2.41, *p* = 0.0161) (Figure 2D), indicating that SMCs express or are covered with above-background HS in AD. Mander’s coefficients revealed that HS overlap with above-background αSMA, not background CD31, is increased in AD patients (Figure S2A). When AD patients with varying CAA severity were grouped, CD31 fluorescence colocalization with HS was not significantly different between the no CAA and mild CAA subgroups (H(2) = 4.53, *p* = 0.1038, mean AD no CAA = 0.51, mean AD mild CAA = 0.48, mean AD severe CAA = 0.55) but significantly increased in the severe CAA group (Z = 2.06, *p* = 0.0392) (Figure 2E). Similarly, αSMA and HS colocalization were increased considerably based on the severity of CAA in AD patients (H(2) = 11.23, *p* = 0.0036, mean AD no CAA = 0.41, mean AD mild CAA = 0.39, mean AD severe CAA = 0.53). AD patients with severe CAA had a significantly increased overlap of HS and αSMA compared to patients with mild CAA (Z = 3.13, *p* = 0.0018) and no CAA (Z = 2.32, *p* = 0.0201) (Figure 2E). Mander’s coefficients indicate that the overlap of HS with EC and SMC channels was increased in the presence of severe CAA (Figure S2B). In summary, these data show that HS in the cerebrovasculature, in both EC and SMC compartments, is elevated in AD patients with severe CAA, revealing a positive correlation between HS level and CAA severity.

### 2.3. Co-Deposition of Cerebrovascular HS with Aβ Is Increased in AD 

**Figure 3:**
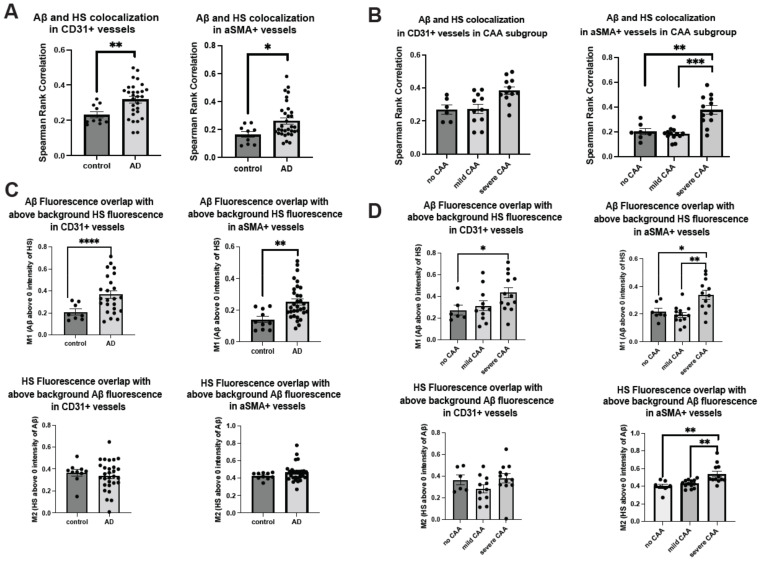
**HS colocalization with Aβ is increased in cerebrovasculature in AD.** HS and Aβ colocalization in the EC and SMC compartments between AD and control (**A**) and CAA-stratified AD subgroups (**B**). Mander’s coefficient analysis to compare the overlap coefficients of the above background staining of HS and Aβ between AD and control (**C**) and between CAA-stratified AD subgroups (**D**). The *p* values for pairwise comparisons are provided. The data are presented as mean ± SD. For significance results, * = *p* ≤ 0.05, ** = *p* ≤ 0.01, *** = *p* ≤ 0.001, **** = *p* ≤ 0.0001.

The co-deposition of HS with Aβ within the cerebrovasculature raises the intriguing question of whether Aβ deposition influences HS levels and HS-Aβ colocalization in the cerebrovasculature or vice versa in AD. Analyzing high-resolution confocal images of HS and Aβ colocalization in both parenchymal and cerebrovasculature, we uncovered elevated HS and Aβ colocalization in the presence of AD in both the EC (control mean = 0.20, AD mean = 0.27, Z = −2.72, *p* = 0.0066) and SMC compartments (control mean = 0.16, AD mean = 0.26, Z = −2.23, *p* = 0.0258) in the Spearman rank correlation analysis (Figure 3A). Similar to the patterns observed in the vascular Aβ deposition in CAA-stratified AD patients, the colocalization of Aβ and HS was significantly increased only in the SMC compartment of AD patients with severe CAA (H(2) = 14.98, *p* = 0.0006, mean AD no CAA = 0.20, mean AD mild CAA = 0.19, mean severe CAA = 0.38) compared to those with no CAA (Z = 3.56, *p* = 0.0004) or mild CAA (Z = 2.75, *p* = 0.006). An increased tendency for CD31 compartment in severe CAA patients was also observed (Figure 3B).

Upon examining the colocalization of HS and Aβ above-background levels using Mander’s M1 and M2 coefficients, we noted that Aβ fluorescence exhibited increased colocalization with above-background HS fluorescence in both EC and SMC compartments of AD patients compared to controls (Figure 3C). However, the overlap of HS fluorescence with above-background Aβ fluorescence remained unchanged in the presence of AD (Figure 3C). In the analysis of CAA subgroups of AD patients, Mander’s coefficients indicated that in the presence of severe CAA, Aβ fluorescence overlap with HS fluorescence increased in both EC and SMC compartments, while the overlapping of HS with Aβ was augmented only in the SMC compartment, not the EC compartment (Figure 3D).

Together, these data show that HS co-disposition with Aβ in cerebrovasculature is increased in AD, especially in the severe CAA subgroup and the SMC compartment. In addition, these data also suggest that the HS level in the SMC compartments is heightened by Aβ deposition.

### 2.4. Polarized HS Expression in Cerebrovasculature Is Reversed in AD with Severe CAA While No Polarization of Vascular Aβ Deposition Is Observed

**Figure 4:**
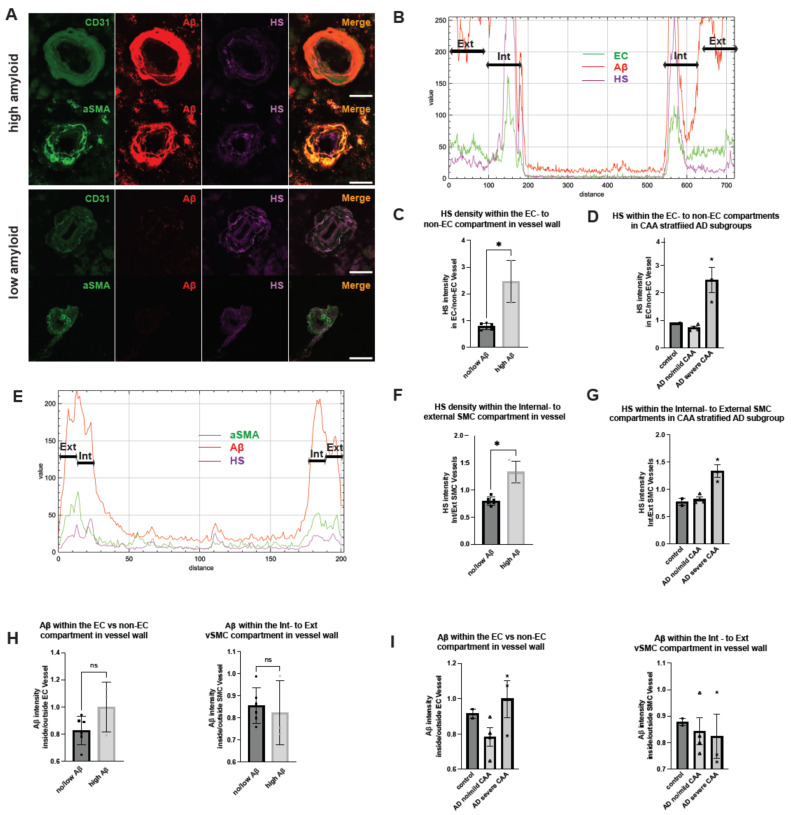
**HS expression and Aβ deposition in different compartments within the cerebrovascular wall in AD.** Representative images of cerebrovasculature with no/low and high CAA are stained for CD31, αSMA, HS, and Aβ (**A**). The immunofluorescence histogram analysis of HS staining in EC to non-EC compartments (**B**–**D**) and the rations of the HS and Aβ staining in the internal (Int)- to the external (Ext) SMC compartments (**E**–**G**). The immunofluorescence histogram analysis of Aβ staining in EC to non-EC compartments (**H**) and the internal to the external SMC compartments (**I**). The data are presented as mean ± SD. For significance results, * = *p* ≤ 0.05; ns, not significant. Scale bars = 25 µm. Jn CAA separated analyses, control patients are denoted with square, AD patients with no/mild CAA are denoted with triangle and AD patients with severe CAA are denoted with asterisk datapoints.

HS is not evenly distributed within blood vessel walls, with much lower HS density in the luminal side, as reported in the skin [51]. The relative expression levels of HS in the various compartments within the cerebrovasculature remain unexplored. By analyzing 2.38 μm stacks of stained tissues, we examined HS expression in different compartments within the cerebrovascular walls (Figure 4A). Employing the immunofluorescence histogram method as reported by Stoler-Barak et al. [52], we initially quantified HS expression in the EC compartment versus the nonendothelial compartment within the vascular wells based on the CD31+ signal (Figure 4B). In control and AD patients with no or mild CAA, the ratios of HS expression in the EC to non-EC compartments were consistently below one, indicating higher HS expression in the non-EC compartment. However, the ratios were increased to more than two in AD patients (low amyloid mean = 0.7835, high amyloid mean = 2.4685, Mann–Whitney U = −1.699824, *p* = 0.0238) (Figure 4C). In CAA stratified groups, only AD patients with severe CAA displayed ratios greater than two, showing the reversal of HS expression polarity in the cerebrovasculature occurs most significantly in this patient subgroup (Figure 4D).

We also analyzed HS expression within the compartment of vascular SMCs. Considering their proximity to endothelial cells, we split the vascular SMC layer equally into internal and external compartments based on the αSMA+ signal (Figure 4E). Analogous to the findings based on EC staining, the ratio of HS expression in the internal to the external compartment was consistently less than one in both normal controls and AD patients with no or mild CAA, and these ratios were higher than one in AD patients (low amyloid mean = 0.7959, high amyloid mean = 1.332, Mann–Whitney U = −0.527625, *p* = 0.0238) (Figure 4F). Similarly, in the CAA-stratified AD subgroups, the internal to external ratios of HS expression within the vascular SMC compartments were greater than one only in the severe CAA subgroup, showing that the reversed polarity of HS expression also occurs within the vascular SMC layer in AD (Figure 4G). These compelling observations underscore the disruption and reversal of polarized HS expression within the cerebrovasculature in AD patients with severe CAA, suggesting a potential role of the reversed HS polarity in contributing to CAA development in these patients.

Of interest, despite increased colocalization with HS, the ratios of Aβ depositions within the EC to non-EC, as well as the ratios of internal to external SMC compartments, were not different between AD patients and controls (Figure 4H). Stratifying patients based on CAA severity yielded no significant differences in Aβ deposition within the endothelial or vascular SMC compartments either (Figure 4I), underscoring that the disrupted and uneven HS expression does not correlate with Aβ deposition within the cerebrovasculature and are most likely consequential events following Aβ deposition.

### 2.5. Male AD Patients Have Lower Parenchymal and Cerebrovascular HS 

**Figure 5:**
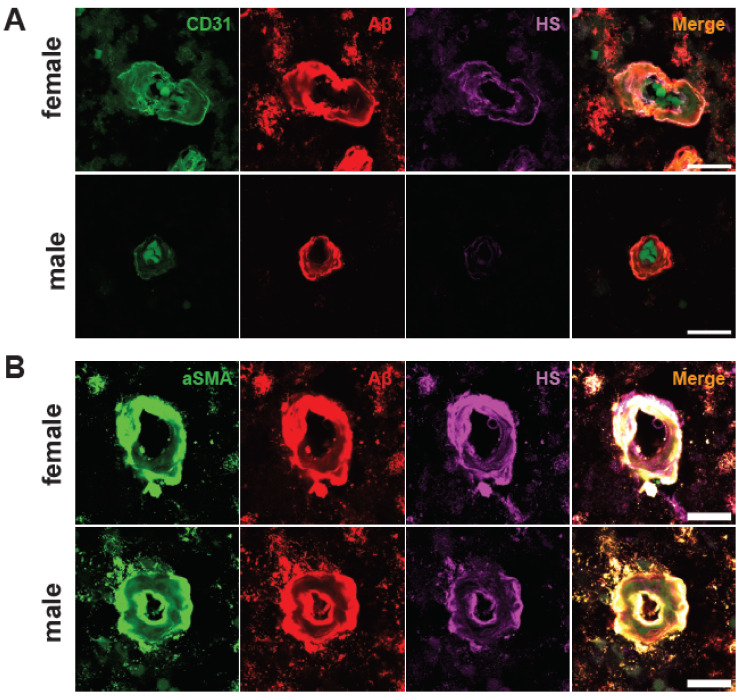

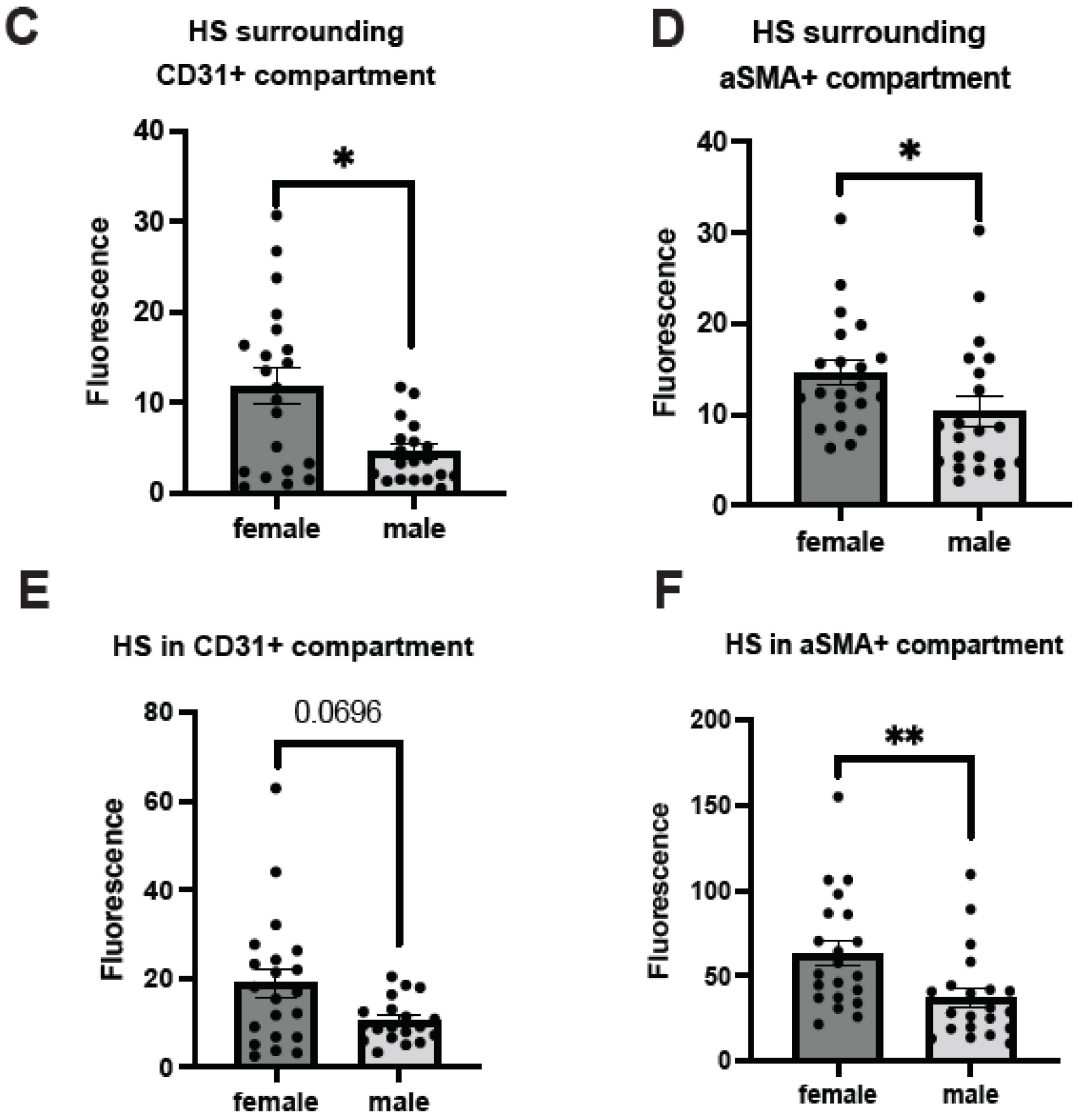
**Cerebrovascular HS expression in male vs. female patients.** Representative male and female brain tissue images stained for CD31, αSMA, and HS (**A**,**B**). Quantitation of HS fluorescence in the compartments surrounding ECs (**C**) and SMCs (**D**), as well as in the compartments of ECs (**E**) and SMCs (**F**). The *p* values for pairwise comparisons are provided. For significance results, * = *p* ≤ 0.05, ** = *p* ≤ 0.01. Scale bars = 25 µm.

There are no significant disparities in cerebrovascular Aβ intensities between patients of different genders (49). However, a notable contrast emerges regarding HS intensity, which is not correlated with other variables, such as ApoE genotype or CAA severity. Specifically, in male patients, HS intensity shows a substantial reduction in the parenchymal regions adjacent to both EC (female mean = 11,58, male mean = 4.37, Z = −2.4376, *p* = 0.0148) (Figure 5A,C) and vascular SMC compartments (female mean = 11.30, male mean = 10.09, Z = −2.3647, *p* = 0.018) (Figure 5B,D). Correspondingly, a parallel diminution is observed in HS intensity within the cerebrovasculature of male patients. Although the EC compartment only presents a tendency of lower HS intensity in males, albeit without achieving statistical significance (female mean = 18.83, male mean = 10.48, Z = −1.8146, *p* = 0.0696) (Figure 5E), the SMC compartment unequivocally exhibits significantly lower HS intensity in male patients (female mean = 62.90, male mean = 37.31, Z = −2.9181, *p* = 0.0035) (Figure 5F). In summary, these data reveal lower cerebral parenchymal and vascular HS expression in male AD patients than in female AD patients.

### 2.6. ApoE4 Correlates with Elevated Cerebrovascular Aβ and a Tendency of Higher HS Expression in AD 

**Figure 6:**
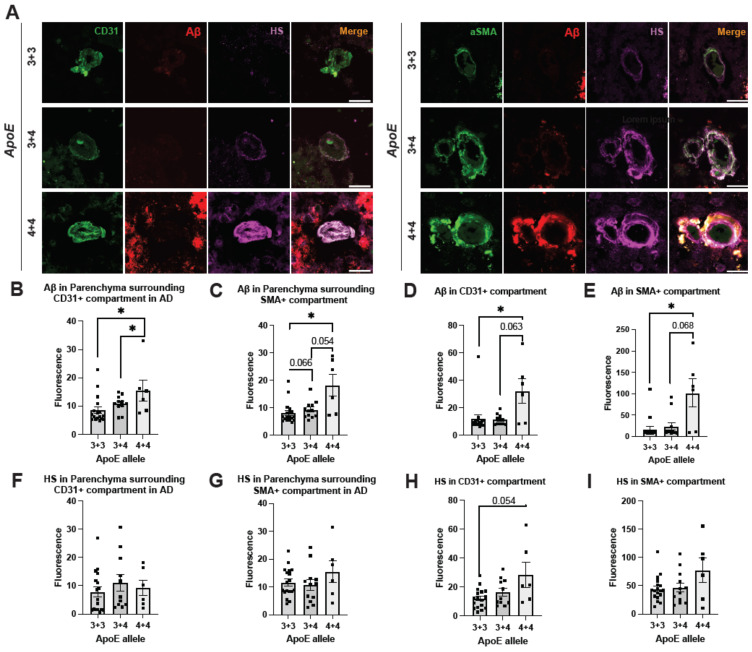
**Cerebrovascular HS expression in AD patients with different ApoE genotypes.** Representative brain tissue images depicting various ApoE genotypes, stained for CD31, αSMA, Aβ, and HS (**A**). Quantitation of Aβ- and HS fluorescence in the compartments surrounding ECs (**B**,**F**) and SMCs (**C**,**G**), as well as in the compartments of ECs (**D**,**H**) and SMCs (**E**,**I**), respectively. The *p* values for pairwise comparisons are provided. For significance results, * = *p* ≤ 0.05. Scale bars = 25 µm.

ApoE4 is a well-established genetic risk factor that significantly increases the likelihood of developing AD, whereas ApoE2 is AD-protective [53]. Currently, the molecular mechanism through which ApoE4 exacerbates AD remains obscure. We stratified our AD samples based on ApoE genotypes, including ApoE3/3, ApoE3/4, and ApoE4/4, and examined if vascular Aβ deposition and HS density correlate with the ApoE genotypes (Figure 6A). We only had three patients who had ApoE 2/3 alleles, and they were not included in the analyses. A positron emission tomography (PET) study did not observe a difference in Aβ deposition based on ApoE genotype [54]. However, we observed a significant grouping effect of ApoE genotype on Aβ deposition in the parenchyma surrounding ECs (H(2) = 7.6374, *p* = 0.022, ApoE3/3 = 8.56, ApoE3/4 mean = 10.83, ApoE4/4 mean = 15.87). Aβ deposition significantly increased with one (Z = 1.9557, *p* = 0.0482) or two (Z = 2.2755, *p* = 0.0229) ApoE4 alleles compared to two ApoE3 alleles (Figure 6B). ApoE4 also has a grouping effect on Aβ deposition in the parenchyma surrounding SMCs (H(2) = 8.9892, *p* = 0.0112, ApoE3/3 mean = 8.10, ApoE3/4 = 9.34, ApoE4/4 = 18.20), and two ApoE4 alleles had significantly higher parenchymal Aβ compared to two ApoE3 alleles (Z = 2.50, *p* = 0.0124), and one ApoE4 allele with one ApoE3 allele had a trending increase of parenchymal Aβ compared to two ApoE3 alleles (Z = 1.84, *p* = 0.0656), as well as a trending increase with two ApoE4 alleles compared to one ApoE3/4 allele (Z = 1.92, *p* = 0.0549) (Figure 6C). When examining cerebrovascular Aβ, the EC compartment of ApoE4/4 carrier had a significant increase of Aβ intensity compared to the ApoE3/3 carriers (Z = 2.0654, *p* = 0.0389, ApoE4/4 mean = 32.33, ApoE3/3 mean = 11.95) and a trending ApoE4 dose effect (Z = 1.8593, *p* = 0.063, ApoE3/4 mean = 11.42, ApoE4/4 mean = 32.33) (Figure 6D). Similarly, the SMC compartment of ApoE4/4 carriers had a significant increase of Aβ intensity compared with ApoE3/3 carriers (Z = 2.500, *p* = 0.0124, ApoE4/4 mean = 101.50, ApoE3/3 mean = 16.95) and a trending ApoE4 dosing effect (Z = 1.8263, *p* = 0.068, ApoE3/4 mean = 23.31, ApoE4/4 mean = 101.50) (Figure 6E). These results support previous findings that indicate that ApoE4 increases vascular Aβ and CAA [55] and may exacerbate AD development.

Previous studies have demonstrated that different ApoE isoforms have different binding affinities to HS, and ApoE4 has a threefold higher affinity for HS than ApoE2 and ApoE3 [56,57]. However, it has not been determined previously if the ApoE isoform is associated with HS expression in AD patients. Examination of the parenchyma surrounding ECs and SMCs has not revealed any difference of ApoE alleles on HS expression in the parenchyma surrounding ECs and SMCs (Figure 6F and G). Interestingly, while examining HS expression in the cerebrovasculature, the EC compartment of ApoE4/4 carriers shows a substantial trending increase (Z = 1.9253, *p* = 0.0542, ApoE 3/3 = 11.92, ApoE4/4 mean = 28.21) (Figure 6H). However, no significant difference in SMC HS expression was detected among the ApoE4/4, ApoE3/4, and ApoE3/3 groups, although the ApoE4/4 group shows a tendency of increased HS density (Figure 6I). These observations suggest a potential association of ApoE4/4 with higher HS expression in the cerebrovasculature in AD.

## 3. Discussion

In AD, Aβ aggregates form neuritic and senile plaques within the brain parenchyma, which serves as a disease hallmark. Additionally, Aβ deposition frequently occurs in the cerebrovasculature, leading to the development of CAA. The development of Aβ deposition in both the parenchyma and cerebrovasculature appears to be driven by impaired clearance mechanisms, which are influenced by factors such as the rate and sources of Aβ generation, its circulation within the interstitial fluid, and the efficiency of perivascular drainage pathways. The initial buildup of Aβ sets the stage for a self-perpetuating cycle, fostering further deposition of parenchymal Aβ plaques and worsening the progression of CAA in AD. In clinical trials of anti-Aβ immunotherapy, approximately 30% of treated patients develop amyloid-related imaging abnormalities caused by microhemorrhages or edema, which cause similar inflammatory responses and leptomeningeal involvement with CAA [12,13,14]. In fact, it is suggested that patients’ high vascular amyloid load may induce amyloid-related imaging abnormalities. A large proportion of AD patients, especially ApoE4 carriers, have some level of CAA in post-mortem examinations [5,58,59]. This phenomenon is thought to result from an overload of Aβ perivascular clearance pathways [12,13,14]. Considerable efforts have been dedicated to unraveling the molecular mechanisms driving Aβ deposition in AD, revealing that the development of vascular and parenchymal Aβ deposition may be governed by distinct regulatory pathways. This is supported by variations in the isoforms of deposited Aβ [60,61,62,63] and specific co-deposited proteins [5] in these two pathological manifestations. 

In AD, HS co-deposits with Aβ in the parenchyma and the cerebrovasculature. In this study, we investigated the expression of HS within the cerebrovasculature and its potential correlation with CAA and AD risk factors in AD patients using immunofluorescence and detailed analyses. Our investigation revealed elevated cerebrovascular HS levels and increased cerebrovascular HS co-deposition with Aβ in AD patients with severe CAA. Particularly noteworthy was the reversal in the polarized expression of HS in the cerebrovasculature among AD patients with severe CAA, contrasting with the absence of a corresponding polarization of vascular Aβ deposition, suggesting that the abnormal cerebral HS expression might be a sequential event following Aβ deposition. Furthermore, male AD patients exhibited reduced levels of parenchymal and cerebrovascular HS compared to females. Additionally, the presence of ApoE4 correlated with heightened cerebrovascular Aβ expression and a tendency toward increased vascular HS expression in AD. In summary, our findings emphasize aberrant HS expression in the cerebrovasculature of AD patients and suggest diverse potential roles of vascular HS in AD pathogenesis, including direct interactions with Aβ and under the influence of AD risk factors, including patient gender and ApoE4 status.

Our research found heightened levels of cerebrovascular HS in AD, aligning with previous findings [24,25,40,49]. For instance, Shimizu et al. noted a 9.3- and 6.6-fold increase in total glycosaminoglycan levels in the hippocampus and the superior frontal gyrus, respectively, in AD brains compared to nondemented controls [24]. In AD brains, HS is more densely concentrated in the thickened basement membrane adjacent to endothelial cells of capillary vessels and in the core of amyloid plaques [24,40]. These observations suggest that the substantial increase in HS in AD brains primarily originates from the capillary basement membrane and senile plaques [24]. In our study, we focused on larger vessels. Our data indicate that the elevation of HS levels in large vessels may also significantly contribute to the marked increase in total HS levels observed in AD patients.

In addition to changes in expression levels, the structure of HS is also altered in AD, as evidenced by modifications in growth factor binding capacities (46) and through the direct chemical analysis of disaccharide and tetrasaccharide compositions [49]. The studies by Wang et al. revealed a significant increase in multiple sulfated disaccharides and a tetrasaccharide containing rare 3S in the human AD frontal cortex [49,50]. Examining whether the structure of cerebral vascular HS is similarly altered in AD would be intriguing. Such analysis could provide a structural framework for better understanding the potential roles of cerebrovascular HS in the development of CAA and AD pathogenesis.

HS exhibits direct binding to Aβ, which is a process that is known to accelerate Aβ aggregation and facilitate Aβ internalization, thereby impacting Aβ metabolism and pathogenesis [35,36,37]. Our study observed increased co-deposition of HS and Aβ in the cerebrovasculature of AD patients, suggesting that heightened interaction may exacerbate pathogenic processes, accelerating detrimental effects on cerebrovascular structure and function and ultimately contributing to AD progression. Moreover, it is established that the most prevalent Aβ isoforms, Aβ40 and Aβ42, preferentially deposit in cerebrovasculature and parenchyma, respectively, although the underlying mechanisms remain elusive [60,61,62,63]. Interestingly, HS exhibits a higher affinity for binding to Aβ40 than to Aβ42 [64,65], and HS has been shown to induce Aβ40, but not Aβ42, to form maltese-cross spherical congophilic plaques identical to those observed in the AD brain [66]. This suggests that the difference in HS-binding affinity may serve as a driving factor for the distinct deposition patterns of Aβ40 and Aβ42 in the AD brain, a phenomenon potentially exacerbated by elevated levels of cerebral vascular HS in AD.

Pathways for clearing soluble Aβ from the brain encompass transport across the blood–brain barrier (BBB), phagocytosis, enzymatic degradation, and perivascular drainage [67]. Animal models and studies examining AD specimens suggest that BBB transcytosis and perivascular drainage are vital mechanisms for Aβ elimination from the brain [68,69,70]. Currently, the roles of cerebrovascular HS in these processes are unknown. Elevated levels of HS may potentially exacerbate Aβ deposition, leading to structural damage to blood vessels and subsequent impairment of vascular function. This could include increased vascular permeability, allowing toxins to access the brain parenchyma and disrupt pathways crucial for BBB- and perivascular drainage-mediated Aβ clearance, thereby promoting AD pathogenesis. Furthermore, our studies revealed a reversal in the polarization of HS expression within the vascular wall. This abnormal polarization might interfere with physiological Aβ clearance pathways, contributing to the deposition of vascular Aβ. Further research aimed at elucidating these potential mechanisms may significantly enhance our understanding of the role of HS in AD.

Sex-based disparities in HS expression under both physiological and pathological conditions have been documented. A study comparing the structural and functional properties of HS chains from male and female adult mouse livers revealed significant differences in chain length and sulfation modifications, with male HS possessing longer chains and female HS exhibiting higher N-sulfation modifications [71]. These structurally distinct forms of male and female liver HS exert differential effects on human mesenchymal cell proliferation and subsequent osteogenic differentiation [71]. In a recent study of a type 2 diabetes rat model, lower HS intensity was reported in male animals, potentially contributing to glucose intolerance and decreased islet insulin secretion in the disease [72,73]. Currently, it remains unknown whether HS levels and structure differ between male and female individuals under normal physiological conditions and in AD patients. In our studies, we observed a tendency for lower HS expression in the cerebrovasculature and parenchyma of male controls compared to females, and this difference became significantly pronounced among AD patients. Additionally, it is noteworthy that women have a higher susceptibility to developing AD, whereas men are more prone to vascular dementia [74]. The disparity in HS expression between males and females could be one of the potential molecular mechanisms underlying the sex-based differences observed in AD and vascular dementia.

ApoE is a secreted protein crucial for regulating lipid transport within the brain. Genome-wide association studies have identified ApoE4 as a major genetic risk factor for AD, whereas ApoE2 is associated with a lower risk than the more common ApoE3 variant [53,75,76,77,78,79]. A growing body of evidence suggests that ApoE4 increases the risk of AD by inhibiting Aβ clearance, promoting Aβ aggregation, and suppressing Aβ cellular uptake and metabolism, although the precise molecular mechanisms remain unclear [80,81,82,83,84,85]. Our study found that ApoE4 correlated with heightened cerebrovascular Aβ deposition and a tendency towards increased vascular HS levels in AD. This observation agrees with early reports that ApoE4 may modulate vascular Aβ deposition [55] and also suggests that ApoE increases vascular HS expression to confer its pathogenic roles in AD.

In our research, we only analyzed a relatively small number of AD specimens and exclusively prefrontal cortex tissues; this limitation has restricted our ability to make certain definitive conclusions, particularly those indicating a strong tendency. It is imperative to conduct additional studies with larger sample sizes, encompassing various AD-related brain regions, and employing both in vitro and in vivo models to deepen our comprehension of the involvement of cerebrovascular HS in AD and CAA.

## 4. Materials and Methods

### 4.1. Human Brain Tissues 

Paraformaldehyde-fixed, cryopreserved postmortem brain tissues from the prefrontal cortex of deceased individuals were obtained from the Emory University Goizueta Alzheimer’s Disease Research Center. All tissues were collected following the ADRC Neuropathology Core protocol approved by the Emory University Institutional Review Board. The samples, which have been summarized in Table 1, consisted of 10 brains from normal controls, 7 from AD patients without CAA, 13 from AD patients with mild CAA, and 12 from AD patients with severe CAA. Each sample was treated as an independent datapoint (n). Neuropathological diagnoses were made according to established diagnostic criteria. Control participants were individuals with no documented history of neurological disorders and no apparent neurodegenerative pathology upon postmortem examination. Comprehensive patient information in Supplementary Table S1 includes details on AD and CAA diagnoses, gender, ApoE genotype, Braak stage, onset and age at death, disease duration, postmortem interval, and associated conditions such as neuritic and diffuse plaques, TAR DNA-binding protein-43 inclusions, cerebral hemorrhage, infarcts, neurofibrillary tangles, and Lewy body dementia.

### 4.2. Immunofluorescence Staining 

The paraformaldehyde-fixed, cryopreserved human brain tissues were frozen, sectioned into 8 µm slices, and mounted onto charged glass slides. These sections underwent immunofluorescent staining using two distinct sets of triple-staining protocols. One set labeled CD31+ EC or αSMA+ SMC, combined with pan anti-Aβ antibody and anti-HS antibody. For the EC triple-staining, an initial antigen retrieval step was performed using 10mM sodium citrate buffer at 95 °C for one hour, while for SMC triple-staining, antigen retrieval was omitted. A consistent staining procedure was applied to all tissue samples, including a one-hour blocking stage, using a mixture of 4% normal goat serum, 1% bovine serum albumin, and 0.05% Triton in PBS. Subsequently, tissues were incubated overnight with primary antibodies, including anti-CD31 (mouse IgG, WM59 clone, concentration 1:75, BioLegend, San Diego, CA, USA, catalog# 303102) and anti-αSMA (goat IgG, dilution 1:200, Novus Biologicals, Centennial, CO, USA, catalog# NB300-978), in conjunction with pan anti-Aβ antibody D54D2 (rabbit IgG, dilution 1:200, Cell Signaling, Danvers, MA, USA, catalog# 8243) and anti-HS antibody 10E4 (mouse IgM, dilution 1:300, Amsbio, Milton, UK, catalog# 370255-1). For the secondary staining phase, Invitrogen, Waltham, MA, USA Alexa Fluor-conjugated antibodies were used at a dilution of 1:700. Specific secondary antibodies included anti-mouse IgG 488 (catalog# A11029), anti-goat IgG 488 (catalog# A11055), anti-rabbit IgG 594 (catalog# A11012), and anti-mouse IgM 647 (catalog# A21042).

### 4.3. Imaging and Image Analyses

The immunostained tissue images were captured using a Leica SP8 confocal laser scanning microscope, with image acquisition performed using the Leica Application Suite X software Version 5.1.0. For each sample, a total of eight images were acquired for analyses of immunofluorescence intensity and colocalization. All samples were visually inspected under the microscope, and representative images were obtained. These images were obtained at a resolution of 1024 × 1024 using a 63× objective lens with 3× optical zoom. For the analyses of HS compartmentalization, four stacks were gathered from each of the 10 samples. These stacks were acquired at a resolution of 1024 × 1024 with a 63× objective and 2× optical zoom, resulting in 2.38 µm stacks composed of eight sequentially acquired images. Following image acquisition, the acquired images underwent analysis using Image J/Fiji (NIH) software Version 2.14.0, and figures were constructed using GraphPad Prism 9 and Adobe Illustrator 2023 Version 27.0 software. Due to imaging parameters set to not oversaturate anti-Aβ intensity in severe CAA cases, more miniscule differences in anti-Aβ intensity were not detected in samples with lower AB burdens. Regions of interest (ROIs) were defined for EC+ or SMC+ areas by applying thresholds on the vascular markers. Areas containing CD31+ white blood cells within blood vessels were excluded from the identified vascular ROIs. In analyzing the parenchyma surrounding the vessels, the portions occupied by the vessels were subtracted from the remaining parts of the images. The Fiji Coloc2 plugin was utilized to estimate colocalization. RGB profile plots were generated using Fiji/ImageJ, and internal and external areas were determined based on the intensity of the vascular markers in cross-sectional vascular images using Microsoft Excel, from which the HS intensities in compartments were deduced. 

### 4.4. Statistical Analyses

In the immunofluorescence intensity and colocalization analyses, each sample is represented by the average of eight images, depicted as a single datapoint on the graphs. The average of four images for HS compartmentalization analyses is illustrated as a single point on the graphs. As the tissue samples were obtained from clinical patients, statistical outliers were retained in the analyses. Given the nonnormal distribution nature of the data, all analyses were performed using nonparametric two-tailed tests. The Wilcoxon two-sample test was employed when comparing two groups, and the results are presented as Z values along with corresponding *p* values. For scenarios involving three or more groups, the Kruskal–Wallis H test was used, and the results include the degrees of freedom, chi-square values, and *p* values. In colocalization analyses, the reported results encompass Spearman’s correlation rank and Manders’ coefficients M1 and M2 values.

## 5. Conclusions

In conclusion, our study reveals a significant association between severe CAA and elevated levels of HS in AD patients. This suggests a potential link between HS expression and the severity of CAA. Furthermore, we observed heightened colocalization of HS with Aβ in these patients, indicating a potential role for HS in the development of CAA. Of particular interest is the discovery of a reversal in the polarization of cerebrovascular HS expression in AD patients with severe CAA, irrespective of Aβ compartmentalization patterns. Additionally, our study identified gender differences, with males exhibiting lower levels of HS compared to females. Moreover, carriers of the ApoE4 allele demonstrated higher expression of cerebral vascular Aβ and a tendency towards increased HS levels in severe CAA cases. These findings underscore the potential intricate interplay between HS, Aβ, and vascular pathology in AD. Importantly, they may provide valuable insights into potential therapeutic strategies aimed at targeting HS in the context of CAA-associated AD pathology. Further research in this area holds promise for the development of novel treatment approaches to combat this devastating neurodegenerative disease.

## Figures and Tables

**Table 1:** Summary of the patients studied.

Patient Diagnosis	Total Number	Gender (M/F)	Age at Death (Ave ± SEM)
**None AD**	**10**	**4/6**	**71.00 ± 5.62**
**AD, no CAA**	**7**	**3/4**	**76.43 ± 3.24**
**AD, mild CAA**	13	8/5	76.46 ± 3.43
**AD, severe CAA**	**12**	**7/5**	**77.75 ± 2.17**

## Data Availability

The research data are available from the corresponding author.

## Supplementary Materials

The supporting information can be downloaded at: https://www.mdpi.com/article/10.3390/ijms25073964/s1.
