# Supplementary material for: Vascular Heparan Sulfate and Amyloid-β in Alzheimer’s Disease Patients
Source: Int J Mol Sci. 2024 Apr 2;25(7):3964. doi: 10.3390/ijms25073964 (PMC11012074; doi:10.3390/ijms25073964)
Supplement: Supplementary file 1 [file ijms-25-03964-s001.zip › ijms-2887843-supplementary.pdf]

International Journal of *Molecular Sciences*

MDPI

**Table S1.** Summary of clinical and pathologic features of the patients studied. LBD: lewy body dementia; DP, diffuse plaques; TDP-43, TAR DNA binding protein-43 inclusions; NFT neurofibrillary tangles.

| AD | CAA | Gender | Sample ID | ApoE | Braak Stage | Age at Onset | Age at Death | Duration (years) | PMI (hours) | Other Diagnoses |
|---|---|---|---|---|---|---|---|---|---|---|
| none | none | F | E05-130 | 3/4 | 0 | | 52 | | 3 | DP |
| none | none | M | E06-41 | 3/3 | 2 | | 57 | | 10 | TDP-43 |
| none | none | F | E06-45 | 3/3 | 0 | | 46 | | 6.5 | |
| none | none | F | E08-101 | 3/3 | 2 | | 78 | | 11.5 | Cerebral hemorrhage |
| none | none | M | E10-142 | 3/3 | 2 | | 94 | | 5.5 | Infarcts, NFT |
| none | none | F | E08-137 | 3/3 | 3 | | 92 | | 15.5 | infarcts |
| none | none | M | E14-06 | | 1 | | 56 | | 12.5 | NFT, ischemia |
| none | none | F | OS2-35 | 3/3 | 1 | | 75 | | 6 | |
| none | none | M | OS3-299 | 3/3 | 2 | | 69 | | 6 | LBD, infarcts, DP |
| none | none | M | E15-106 | | 2 | | 61 | | 6 | |
| AD | none | F | E05-90 | 3/4 | 6 | 69 | 76 | 7 | 8 | LBD |
| AD | none | M | E08-104 | 3/3 | 5 | 79 | 89 | 10 | 22 | LBD, TDP-43 |
| AD | none | M | E11-139 | 2/3 | 6 | 55 | 62 | 7 | 6 | |
| AD | none | F | OS3-300 | 4/4 | 6 | 60 | 75 | 15 | 12 | |
| AD | mild | F | E06-190 | 3/4 | 6 | 80 | 93 | 13 | 5 | |
| AD | mild | M | E16-21 | | 6 | 55 | 62 | 7 | 3 | |
| AD | mild | M | E07-36 | 3/3 | 5 | | 91 | | 22 | |
| AD | mild | M | E07-38 | 3/4 | 6 | 70 | 77 | 7 | 12 | TDP-43 |
| AD | mild | F | E07-69 | 3/4 | 6 | 53 | 58 | 5 | 6 | |
| AD | mild | M | E08-41 | 3/4 | 6 | 76 | 84 | 8 | 12 | infarcts |
| AD | mild | F | E08-108 | 3/4 | 4 | 76 | 94 | 18 | 4 | TDP-43, sclerosis, Rosenthal fibers |
| AD | mild | F | E10-33 | 3/3 | 6 | 52 | 62 | 10 | 6 | LBD, TDP-43 |
| AD | mild | F | E16-110 | | 6 | 64 | 77 | 13 | 14 | TDP-43 |
| AD | severe | F | E05-37 | 4/4 | 6 | 71 | 86 | 15 | 6.5 | LBD, infarcts |
| AD | severe | M | E06-155 | 2/3 | 6 | 56 | 67 | 11 | 8.5 | LBD |
| AD | severe | M | E07-84 | 3/4 | 6 | 67 | 72 | 5 | 16 | microinfarcts |
| AD | severe | F | E08-53 | 3/3 | 6 | 70 | 78 | 10 | 8 | TDP-43 |
| AD | severe | F | E09-65 | 4/4 | 5 | 77 | 85 | 8 | 10 | Infarcts |
| AD | severe | M | E09-126 | 3/4 | 6 | 63 | 68 | 5 | 17 | TDP-43 |

| | | | | | | | | | | |
|---|---|---|---|---|---|---|---|---|---|---|
| AD | severe | M | E11-97 | 3/3 | 6 | 60 | 74 | 14 | 2.5 | |
| AD | severe | F | E13-116 | 3/4 | 6 | 67 | 77 | 10 | 10 | sclerosis |
| AD | severe | M | OS0-40 | 4/4 | 5 | 83 | 88 | 5 | 4.25 | TDP-43, infarcts |

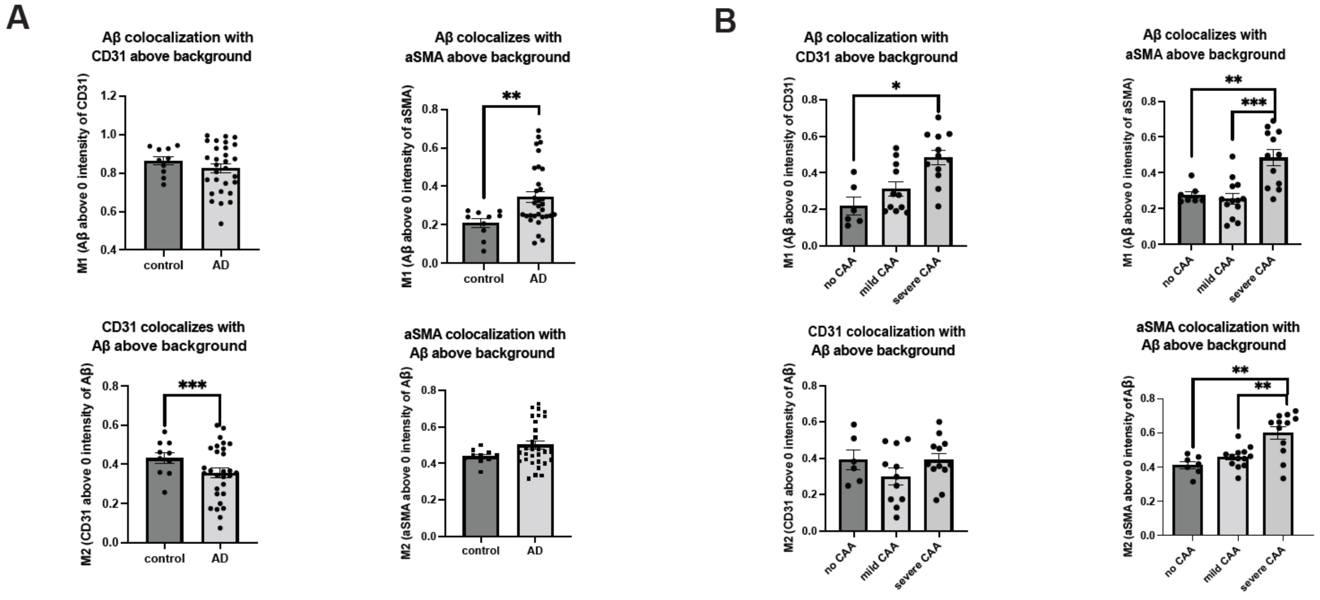


**Figure S1. Mander's M1 and M2 coefficient analysis of the overlap of Aβ with CD31 or αSMA.** The overlap between Aβ and above-background CD31 fluorescence remains unaffected (M1), while CD31 fluorescence overlap with above-background Aβ fluorescence (M2) is elevated in the presence of AD (A). Aβ fluorescence overlap with above-background αSMA fluorescence (M1) is increased in the presence of AD, and there is a tendency of increase for αSMA fluorescence overlap with above-background Aβ fluorescence in AD as well (A). In the CAA-stratified AD groups, only the severe CAA subgroup exhibits an increased Aβ fluorescence overlap with above-background CD31 and αSMA fluorescence (M1s). Meanwhile, αSMA fluorescence shows an increase in overlap with above-background Aβ fluorescence (M2), and CD31 fluorescence overlapping with Aβ fluorescence does not exhibit a significant change (B). The p values for pairwise comparisons are provided. The data are presented as mean ± SD. For significance results, * = $P \leq 0.05$, ** = $P \leq 0.01$, *** = $P \leq 0.001$. For the comparison of CAA group effect analyses, the Kruskal Wallis H test, and Chi Square values are reported.

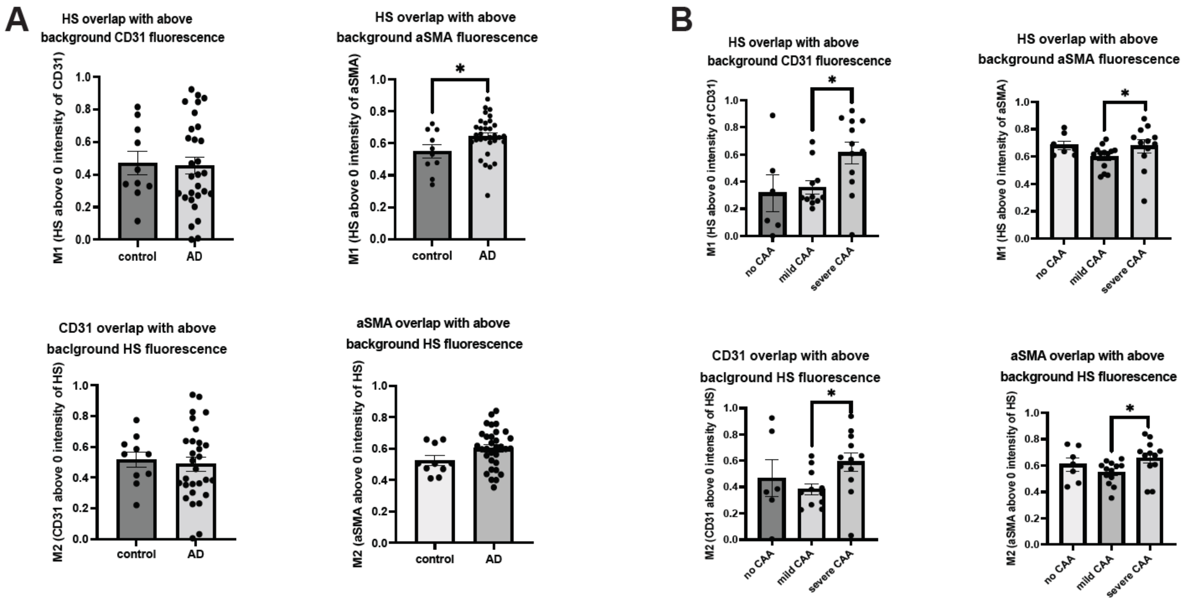


**Figure S2. Mander`s coefficient analysis.** The comparison of overlap coefficients for the following pairs in EC- and SMC compartments between AD and control: above background HS-CD31 staining, above background CD31-HS staining, above background HS-$\alpha$SMA staining, and above background $\alpha$SMA-HS staining (**A**). A similar comparison between CAA-stratified AD subgroups (**B**). The data are presented as mean ± SD. For significance results, * = P ≤ 0.05.
